# Sudden Sensorineural Hearing Loss and Why It’s an Emergency

**DOI:** 10.7759/cureus.21418

**Published:** 2022-01-19

**Authors:** Wynnie W Jung, Carl Hoegerl

**Affiliations:** 1 Neurology, Liberty University College of Osteopathic Medicine, Lynchburg, USA; 2 Neurology, Centra Health System, Lynchburg, USA; 3 Internal Medicine and Neurology, Liberty University College of Osteopathic Medicine, Lynchburg, USA

**Keywords:** conductive hearing loss, acoustic neuroma, acoustic schwanomma, unilateral hearing loss, hearing loss, sudden sensorineural hearing loss(ssnhl)

## Abstract

This review shows the importance of sudden sensorineural hearing loss (SSNHL) as a significant cause of hearing loss that often goes under-recognized, especially by primary care physicians. Contrasted with conductive hearing loss, SSNHL involves inner ear damage. This work reviewed the diagnostic methods and treatments of SSNHL in the U.S. and Canada, including treatment options. It is imperative that primary care physicians and providers be able to recognize this condition early so that treatment may be initiated without delay as hearing loss can become permanent if not managed immediately.

## Introduction and background

Sudden sensorineural hearing loss (SSNHL) is an otological condition that develops rapidly in a patient over the course of hours to days and is considered an ENT emergency [1]. The patient may notice sudden hearing loss that develops quickly or they may wake up with a hearing loss. There may be associated tinnitus or a sense of "fullness" in the ear that they have not noticed before [1]. If not treated quickly, the effects of the condition can become permanent and come with several comorbidities [2]. The incidence of SSNHL is approximately 5-27 per 100,000 people annually, with incidences peaking in those 50 to 60-years-old [1,2]. SSNHL is defined as a rapid onset of ≥30 dB hearing loss across three contiguous frequencies in 72 hours or less [2-5].

Contrasted from conductive hearing loss, SSNHL involves inner ear or nerve damage with unilateral ear involvement more likely [1,2,4]. Conductive hearing loss usually involves some obstruction of the outer or middle ear such as an infection or cerumen impaction [1]. The presence of vertigo signifies a lower prognosis in patients with SSNHL and greater than 60-years-old or less than 15-years-old [1,2]. Better prognosis occurs in those with low-frequency loss, severe hearing loss at presentation, and early commencement of treatment [1].

Although most cases of SSNHL are idiopathic, some researchers have theorized, among others, a vascular etiology, viral infection, viral reactivation, otologic, traumatic, or neoplasm as the origin [2,6,7]. Viral infections can lead to cochlear inflammation and/or damage to critical inner ear structures [2,3]. Hearing loss due to intravascular insult and viral infections are severe and permanent, but a majority of idiopathic SSNHL is reversible, making it crucial for primary care physicians to treat this problem as soon as possible [2]. There is also a variable connection, ranging from 0-48%, between vestibular schwannoma and SSNHL that often warrants MRI scans [2]. Controversy still surrounds the treatments for SSNHL as some patients recover spontaneously while others require the use of steroids. This article will examine the clinical presentations of patients with SSNHL and methods to confirm the diagnosis and treatment.

## Review

Diagnosis

Symptoms

SSNHL is an emergency case that can lead to permanent hearing loss if not treated quickly and properly [7]. Many patients with SSNHL have a history of clear and sudden change in hearing or of awakening with new hearing loss, complaining of having a “blocked ear” [1]. Many patients just think that they have an ear infection and don’t go to the doctor right away. These symptoms resemble those in patients with conductive hearing loss (CHL), making it crucial to differentiate between the two early on to ensure proper management. CHL is the most common type of hearing loss and involves problems affecting the external and middle ear, while SSNHL involves the inner ear [1]. Tinnitus and dizziness are non-specific and may help to differentiate between CHL and SSNHL [1,3]. In one study evaluating management of SSNHL among primary care physicians, the authors found that nearly all participants would warrant a referral to an otolaryngologist, which is contingent on whether the referring physician can recognize SSNHL [4]. Other exams discussed later in this article can be performed to confirm SSNHL and may help cut down on the time to proper diagnosis and treatment.

Evaluations

Tuning fork tests, audiograms, otoscopy, and thorough case history is essential in making a proper diagnosis of SSNHL [1,4]. A crucial first step for physicians is to differentiate between SSNHL and other hearing issues. Due to the nature of SSNHL, patients will almost always have normal external auditory canals and tympanic membranes, resulting in a normal otoscopic exam [3,4].

Tuning fork tests, specifically the Weber and Rinne tests, can be used to differentiate between CHL and SSNHL [1,4]. A 512 Hz tuning fork provides the most reliable response and should be used to perform these tests, but a 256 Hz tuning fork can be used as an alternative [1,8]. The Weber test detects lateralization in hearing, while the Rinne test determines whether air conduction is greater than bone conduction (normal/positive) [8]. The Weber test is highly sensitive and capable of indicating the affected ear, which is verified by the Rinne test to determine the presence (negative) or absence (positive) of CHL in the affected ear [1,4].

Despite the practicality of tuning fork tests, only 63.5% of primary care physicians in a study evaluating management of SSNL used these tests [4]. Many participants relied on audiological evaluation, which can delay treatment for patients as physicians wait for test results. Although the Weber and Rinne tests do not replace audiological assessments, they can help direct physicians' next steps. If physicians are not confident in performing tuning fork tests, they can use the Hum test as an alternative to the Weber test [4].

Although more time-consuming than tuning fork tests, standard pure tone audiometry is the most reliable method in differentiating between CHL and SSNHL [1,7]. Pure tone audiometry contains diagnostic and prognostic values, capable of determining the degree and type of hearing loss [1,2,9]. Negative prognostic indicators present with a flat or down-sloping audiogram shape, whereas more positive prognostic indicators present as low- or mid-frequency hearing loss configurations [4].

As mentioned earlier, researchers have discovered connections between vestibular schwannoma and hearing loss. One study found that 95% of patients with vestibular schwannoma suffered from hearing loss, and 7-20% of those patients had SSNHL [1]. This makes radiographic evaluation of the internal auditory canal a major component in diagnosing SSNHL. Magnetic resonance imaging (MRI) with gadolinium contrast of the internal acoustic meatus and brain, coupled with cerebellopontine angles for tumors (specifically vestibular schwannomas and meningiomas) is used by many physicians to diagnose unilateral SSNHL [1,2,4]. 

Auditory brainstem response testing (ABR) can be a useful alternative when MRI is not available or contraindicated. The sensitivity of ABR compared to MRI in diagnosing tumors is lower [2]. Although a useful substitute, ABR cannot exclude vestibular schwannomas from all SSNHL patients because sufficient residual hearing is required for an ABR response [2].

Much controversy surrounds the need for lab work in patients with SSNHL. For example, in one study, 17.3% and 15.4% of family physicians ordered lab work and CT scans, respectively [4], but some guidelines suggest that for sudden hearing loss such orders are unnecessary for initial treatment of SSNHL [4]. Certain blood tests can help establish a cause of SSNHL in some cases, such as TSH and markers of inflammation and autoimmune disease (ESR), but may not always be needed [1]. Almost half of the patients with idiopathic SSNHL also have hyperglycemia, thus elevated blood glucose can be seen in SSNHL patients [2]. In cases that involve neurological issues but unclear diagnosis, physicians may consider ordering cerebral spinal fluid testing to obtain cell counts, Lyme antibody titers, and tests for other infectious agents [2]. Although useful in determining the root cause of SSNHL in some patients, treatment can be started without such tests in most cases.

Treatment

Disagreement still occurs around the treatment plan for patients presenting with SSNHL. On average, 46.7% of patients with idiopathic SSNHL spontaneously recover without treatment within two weeks of onset, yet other studies have found a significantly higher rate of recovery and improved hearing recovery in patients treated with steroids compared to placebo [1,2,4,9]. Authors of another study determined no significant differences between steroids and placebo in terms of speech frequency and high-tone hearing levels [9]. Authors who undertook a systematic review of existing studies were unable to determine the value of steroids to improve hearing and reduce tinnitus in patients with idiopathic SSNHL [6].

A majority of U.S. otolaryngologists, however, reported the use of oral steroids to treat idiopathic SSNHL as part of initial management with a small percentage using intratympanic steroids based on their ability to decrease inflammation and edema [1,2,6]. Chances of complete hearing recovery, especially for those who start treatment within seven days of onset, have significantly better hearing outcomes compared to those who start later [2,4,7]. Most patients are treated with a seven to 14-day course of oral steroids, and tapering is not required for shorter courses [1].

Some physicians have given intratympanic (IT) steroid injections as primary therapy for SSNHL, in combination with oral steroids, or as salvage therapy for patients who failed the initial course of oral steroids [10]. IT injection of corticosteroids is not inferior to systemic steroids with thresholds less than 70 dB hearing loss, with a significantly greater number of patients having an improvement of at least 30 dB in hearing [2,5,9]. Researchers have concluded that IT steroid injections either after an initial 10-day course of systemic steroids or combined with systemic steroids can result in greater rates of recovery and increased hearing improvement in patients versus the use of systemic steroid or IT steroid injections alone [5,9]. Researchers recommended an oral intake of 60 mg/day of prednisone for 7-10 days coupled with 24 mg/mL of dexamethasone IT steroid injection over two weeks [5]. Injections should go through the round window to ensure higher steroid concentrations are maintained over a longer period of time [3,5]. COVID-19 infections have been shown to cause harmful effects on cochlear hair cell functions, and researchers suggest the use of IT steroid injections as controversy surrounds the use of systemic steroids in COVID-19 patients [3]. In addition, IT may be better tolerated by patients that have co-morbid conditions such as diabetes.

Due to the viral origin of a few cases of SSNHL, some physicians have prescribed antivirals alongside steroids or as the main treatment, but several studies have found no statistically significant improvement in the outcome of patients’ SSNHL after the use of antivirals [4,7,9]. Thus far, the use of antivirals as a treatment of idiopathic SSNHL has not been proven effective by researchers [7]. In addition, antivirals have been reported to cause unwanted side effects [1,4].

Recently, hyperbaric oxygen (HBO) therapy has been used on patients with SSNHL to improve the rate and quality of recovery. SSNHL can be a consequence of impaired blood flow to the cochlea, causing hypoxia in the perilymph and structures within that to require a high oxygen supply [4,10,11]. Patients in a case study who were given HBO therapy after 17 days of oral corticosteroids showed complete recovery of hearing confirmed by pure tone audiometry along with statistically significant hearing gains across all frequencies. SSNHL patients who experienced tinnitus showed the greatest hearing improvements, but no data shows the benefits of HBO therapy beyond 10-20 sessions. When combined with large-dosed steroid therapy, the overall effectiveness of HBO can vary from 11-80% with the most favorable prognoses in patients experiencing a medium or deep hearing loss (>41 dB) and whose treatments were implemented within 14 days of onset. A common side effect of HBO therapy can include bilateral nearsightedness (myopia) that normally returns over the course of a few weeks to three months from the date of completion without any intervention [10].

To ensure successful treatment, audiometric evaluation is recommended for all patients with SSNHL after completion and within six months of treatment [10]. Some patients may also see improvements in their concomitant tinnitus with successful treatment [4]. If patients still experience residual or permanent hearing loss and/or tinnitus after completion of treatment, physicians should counsel patients about audiologic rehab and other supportive methods [10]. 

## Conclusions

The bottom line for this review article is that both patients and providers need to know the importance of being able to recognize and identify sudden sensorineural hearing loss (SSNHL) and the emergency that it is. It is imperative that providers be able to recognize this early so that it may be treated quickly and properly. Too many times it is easy to assume that a patient may have just a simple ear infection, allergies, or something else when in actuality it may be SSNHL. It is important, especially for the primary care provider, to rule out potential causes of conductive hearing loss (ie cerumen impaction) and if SSNHL is suspected, then the patient should be seen by the otolaryngologist or other provider as soon as possible. 
